# Arbuscular mycorrhizal fungi reduce arsenic uptake and improve plant growth in *Lens culinaris*

**DOI:** 10.1371/journal.pone.0211441

**Published:** 2019-05-16

**Authors:** Mohammad Zahangeer Alam, Md. Anamul Hoque, Golam Jalal Ahammed, Lynne Carpenter-Boggs

**Affiliations:** 1 Department of Environmental Science, Faculty of Agriculture, Bangabandhu Sheikh Mujibur Rahman Agricultural University (BSMRAU), Gazipur, Bangladesh; 2 Department of Soil Science, Bangladesh Agricultural University (BAU), Mymensingh, Bangladesh; 3 Department of Crop and Soil Sciences, Washington State University (WSU), Pullman, WA, United States of America; 4 College of Forestry, Henan University of Science and Technology, Luoyang, PR China; The University of Newcastle, AUSTRALIA

## Abstract

Arsenic (As) is a carcinogenic and hazardous substance that poses a serious risk to human health due to its transport into the food chain. The present research is focused on the As transport in different lentil genotypes and the role of Arbuscular Mycorrhizal Fungi (AMF) in mitigation of As phyto-toxicity. Arsenic transport from soil to root, shoot and grains in different lentil genotypes was analyzed by flow injection hydride generation atomic absorption spectrophotometry. AMF were applied for the reduction of As uptake as well as the improvement of plant growth in lentil genotypes. Arsenic phyto-toxicity was dose-dependent as evidenced by relatively higher shoot length, fresh and dry weight of root and shoot in 5 and 15 mgkg^-1^ As-treated lentil plants than that in 100 mgkg^-1^ As-treated lentil. Arsenic accumulation occurred in roots and shoots of all BARI-released lentil genotypes. Arsenic accumulation in grains was found higher in BARI Mashur 1 than other lentil genotypes. AMF treatment significantly increased growth and biomass accumulation in lentil compared to that in non-AMF plants. Furthermore, AMF effectively reduced the As concentrations in roots and shoots of lentil plants grown at 8 and 45 mgkg^-1^ As-contaminated soils. This study revealed remarkable divergence in As accumulation among different BARI-released lentil genotypes; however, AMF could reduce As uptake and mitigate As-induced phyto-toxicity in lentil. Taken together, our results suggest a great potential of AMF in mitigating As transfer in root and shoot mass and reallocation to grains, which would expand lentil cultivation in As-affected areas throughout the world.

## Introduction

Arsenic (As) is a natural but hazardous element present in rocks, soils, water, air, and biological tissues [1]. In recent years, research on the occurrence, distribution, origin, and mobility of As in soils through natural, geochemical and biological processes has remarkably increased [2]. According to the U.S. Agency for Toxic Substances and Disease Registry (ATSDR) priority list of hazardous substances, As has been designated as the number one hazardous substance in the United States [2]. Moreover, As contamination has been reported worldwide, particularly in Argentina, Australia, Bangladesh, Chile, China, Hungary, Mexico, Peru, Thailand, and Vietnam [3]. However, the most severe As contamination to surface soil, water, and humans is currently in Asia, particularly Bangladesh, [3] West Bengal and India [4].

Arsenic has been recognized as a carcinogenic substance based on its chemical and physical forms as well as concentration and duration of exposure [5]. Chemically, it exists as organic and inorganic species. The main sources of As are arsenic sulphide (As_2_S_2_), arsenic tri-sulphide (As_2_S_3_) and arsenopyrite or ferrous arsenic sulphide (FeAsS_2_) [1]. Inorganic As has two main oxidation states i.e., trivalent arsenite As(III), and pentavalent arsenate As(V). The inorganic forms of arsenate As(V) and arsenite As(III)) are usually dominant in As contaminated soil. The arsenite As(III) in the presence of herbicides and pesticides is oxidized into As(V) [6]. Trivalent arsenite is 60 times more toxic than arsenate [1].

Arsenic causes highly toxic effects on metabolic processes of plants, mitotic abnormalities, leaf chlorosis, growth inhibition, reduced photosynthesis, DNA replication, and inhibition of enzymatic activities [7,8,9]. For instance, root and leaf elongation of the mesquite plant *(Prosopis juliflora x P*. *velutina*) decreased significantly with increasing As (III) and As (V) concentrations [10]. [11] reports that As contaminated water leads to accumulation in the soil, which is then transported into edible parts of food crops. Arsenite As(III) and arsenate As(V) both are present in wheat crops due to accumulation from soils to shoots and grains [6]. In addition, the extensive use of pesticides, fertilizer, groundwater and industrial wastewater for irrigation purposes in crop fields has resulted in elevated levels of As in soils, and thus increased As uptake into edible parts of rice, lentil and vegetables [3]. Consequently, many food crops have become hazardous including Lentil, which is a major leguminous crop across the world. These crops are an excellent source of protein, minerals and vitamins for human nutrition [12]. Similarly, chronic exposure of As has led to unacceptable As levels in samples of soils, water, vegetables and cereals. Subsequently, high Average Daily Dose (ADD) from the environment and low excretion could result in As toxicity to humans from lentil crops as well as from other food crop cultivation in As contaminated soils [13]. Furthermore, As carcinogenicity has caused serious health diseases, such as lung and skin cancers, and possible damage to liver and kidneys as well. Noncancerous health effects of As exposure include diabetes, chronic cough, and cardiovascular and nervous system collapse http://www.hindawi.com/journals/tswj/2014/921581/ - B7[14].

Currently, Bangladesh is the second largest area of As contamination in the world. Bangladesh is facing a serious public health threat, with 85 million people at risk of As contamination in drinking water and food crops. In addition, 85–95% of rice, lentil and vegetable crops are contaminated by As, which poses a serious threat to human and livestock health [1,7]. Therefore, it is imperative for the mitigation of As in crop plants. One possible solution includes Arbuscular Mycorrhizal Fungi (AMF), which establishes a mutualistic symbiotic relationship with the majority of terrestrial plant including lentil crops [15,16,17]. AMF are actively involved in As accumulation, and AMF colonization affects the concentration of As, Cd, Zn, and Pb in shoots and roots [16,18,19,20]. The effect of AMF on element uptake can, vary largely, depending on plant species/cultivar and metal concentration in the soil, but also on AM fungal species and isolates [18]. In aerobic soils the main form of As is arsenate As(V). In this form As mimics phosphorus (P), and can be taken up by lentil plants and AMF by normal P uptake mechanisms [21]. In this circumstance, mycorrhizal symbioses are significantly highlighted because they are formed by 90% of higher plants, often with increased uptake of phosphate (P) compared with non-mycorrhizal (NM) counterparts [22]. It is clear that the association of AMF inoculation with food crops might reduce As uptake by various mechanisms [3,23]. The high proportion of inorganic species of As (As*i*) is of particular concern to the human carcinogen through the protein sources of lentil crops. Lentil is one of the major leguminous crops in the world. The future of agriculture will depend increasingly upon legume crops because of production of high energy and protein for human and animal health nutrition. Therefore, As mitigation technique is very much a necessity for lentil crops as well as other crops. The present research focused to lentil varietal selection against As and its impact on lentil’s biomass production and health risk associated with the consumption of contaminated grains. This research also highlights the reduction of As accumulation in roots, shoots and grains using the AMF. It hypotheses that this research will provide a solid foundation for the exploration of low As accumulator lentil genotypes that could supply As free grains for the consumption to humans.

## Materials and methods

### Experiment 1: Arsenic uptake in root and shoot of lentil genotypes

#### Description of soil sampling areas

Arsenic-contaminated soils were collected for the pot experiment from Mathchar (As concentration 15 mgkg^-1^) (Faridpur), Bangladesh Jute Research Institute (BJRI) area (As concentration 8 mgkg^-1^) (Faridpur) and Bangabandhu Sheikh Mujibur Rahman Agricultural University (BSMRAU) research field (As concentration 5 mgkg^-1^) (Gazipur), Bangladesh in 2015. The Global Positioning System (GPS) are 23^0^35.38969', 24^0^2.17859', & 23^0^35.97636' Latitudes and 89^0^48.69921', 90^0^23.83393', & 89^0^46.7586' Longitudes in the soil sampling locations of BJRI (Faridpur), BSMRAU (Gazipur) and Mathchar (Faridpur), respectively. The soil of the study areas is silty loam in the agro-ecological zone of the Old Meghna Estuarine floodplain of Bangladesh, which falls under the order of Inceptisols according to the USDA (United States Department of Agriculture) soil classification.

#### Collection of lentil genotypes

Seven out of eight lentil varieties developed by Bangladesh Agricultural Research Institute (BARI) were procured from the pulse research center of BARI for the current study. These lentil varieties are BARI Mashur1, BARI Mashur 2, BARI Mashur 3, BARI Mashur 4, BARI Mashur 5, BARI Mashur 6 and BARI Mashur 7. The average yields of these varieties are 2 to 2.3 tons per ha. Total duration required from seed to maturity is about 100 to 105 days [7]. The production season of these lentil genotypes is from November to February in Bangladesh. These varieties were chosen in this study based on their height, life cycle, growing season, and yield.

#### Samples preparation

Before land preparation, soil samples were collected from As-contaminated regions in Bangladesh using a soil auger to a depth of 15 cm and brought into the Department of Environmental Science at Bangabandhu Sheikh Mujibur Rahman Agricultural University (BSMRAU). Before sowing lentil seeds in a pot, initial soil samples of 250–300 (g) were taken from each composite through the guidelines of BARC [24]. The soil was air dried at room temperature in the laboratory. Samples were then ground and sieved with a ≤250 μm mesh and preserved in polythene bags with proper labeling. Vermi-compost samples were also prepared for chemical analysis as well as soil samples. On the other hand, seeds, roots and shoots of lentil genotypes were kept in an oven for drying at 55°C for 72 hours. Samples were then ground using coffee grinder and liquid nitrogen and sieved with ≤250 μm mesh for chemical analysis. Meanwhile, dry weight of lentil seeds was measured by digital electrical balance after drying in an oven.

#### Analysis of pH, N, P, K, S in soil, water, vermi-compost

The pH of soil, irrigation water and vermi-compost were determined by glass electrode pH meter [25]. Total N percentage of the soil and vermi-compost were determined by Kjeldhal systems [25]. Available P of the soil and vermi-compost were determined by Olsen method [26]. Exchangeable K of the soil and vermi-compost were estimated by Ammonium Acetate Extraction method [25]. Available sulfur of the soil and vermi-compost were determined by turbidimetrically as barium sulfate method [27] (Table 1).

**Table 1:** Dry weight and chemical properties of lentil seeds, soils, vermi-compost and water samples.

Materials	Grain weight (g)	Dry weight (g)	As (mgkg^-1^)	pH	Total nitrogen %	Available phosphorus (mgkg^-1^)	Exchangeable potassium (mgkg^-1^)	Available sulfur (mgkg^-1^)
Distilled water	..	..	0	7.18	-	-	-	-
Irrigation water	..	..	0.0208	7	-	-	-	-
BARI Mashur 1	10	9.47	0	..	-	-	-	-
BARI Mashur 2	10	9.5	0.00045	..	-	-	-	-
BARI Mashur 3	10	9.51	0.00485	..	-	-	-	-
BARI Mashur 4	10	9.43	0.05575	..	-	-	-	-
BARI Mashur 5	10	9.44	0	..	-	-	-	-
BARI Mashur 6	10	9.49	0	..	-	-	-	-
BARI Mashur 7	10	9.53	0	..	-	-		-
Vermi-compost	..	..	2.6882	6.75	1.23 (12300mgkg^-1^)	57.71	150	698.04
BJRI Soils (t_3_)	..	..	8.2997	7.93	0.057 (570mgkg^-1^)	14.41	120	9.615
BSMRAU soils (t_1_)	..	..	5.2237	7.74	0.11 (1100 mgkg^-1^)	20.68	124	23.07
Mathchar soil (t_2_)	..	..	14.6337	7.73	0.086 (860mgkg^-1^)	9.177	128	2.884

#### Nutrient augmentation in soils by fertilizers for growing lentil genotypes

Collected soil samples were ground uniformly for sowing of lentil seeds. One and half kilogram of ground soils with 200g vermi-compost were mixed together in each pot. According to the recommendations of the Bangladesh Agricultural Research Institute (BARI), Urea 225kgha^-1^, TSP 450kgha^-1^ and MOP 175 kgha^-1^ were incorporated into the soil in each pots. Total nitrogen (46%), phosphorus (20%) and potassium (51%) were added in each experimental pot with the synthetic fertilizers. Before sowing of lentil seeds, Vitavex 200 fungicides (1gL^-1^ water) used as seed treating chemical. Clay pots size 6″/6″were used in this experiment. All types of input materials purchased from the local market of Bangladesh for this pot experiment. Then 7–10 lentil seeds of each variety sowed in each pot during the first week of November in 2015.

#### Treatments and replications

Based on the As concentration in soils, three samples were selected as treatment for this pot experiment. These treatments included t_1_ = arsenic concentration 5 mgkg^-1^ (BSMRAU soil), t_2_ = arsenic concentration 15 mgkg^-1^ (Mathchar soil- Faridpur) and t_3_ = arsenic concentration (8+92) = 100 mgkg^-1^ (BJRI soil- Faridpur). The concentration of As in background soils (8 mgkg^-1^) was increased to 100 mgkg^-1^ through the addition of sodium arsenate (Na_2_HAsO_4_.7H_2_O) as a source of arsenic. A 0.3831 g sodium arsenate was used for each kg of soil in the pots to get the required concentration of arsenic (100 mgkg^-1^). Five replications with seven lentil varieties were used in this pot experiment and the total number of pot was 105.

#### Shoot length, root and shoot mass of lentil genotypes grown in As contaminated soils

At random, average shoot lengths were measured using a measuring tape (cm) at week 3 in each treated pots. At this time point, three lentil seedlings were thinned out from each As treated pot. Fresh weights were taken of each sample using electrical balance (g). Average dry weight of roots and shoots were measured separately after harvesting of lentil seedlings from each As treated pot during week nine. All samples were dried in an oven at 55°C for 72 hours towards the digestion of samples for the determination of As uptake in root and shoot of lentil crops from As contaminated soils.

### Experiment 2: Arsenic uptake in lentil pods from As-contaminated field soils

Simultaneously, **s**even lentil genotypes were sown on 12 November 2015 in As contaminated field soils. For this field experiment, 10 x 5-meter sizes of seven plots were prepared at BSMRAU research fields. BARI released seven lentil genotypes sown in seven plots separately. All plots in soils were with an As concentration of 5 mgkg^-1^. Recommended doses of fertilizers Urea 225kgha^-1^, TSP 450kgha^-1^ and MOP 175 kgha^-1^ were applied in this field experiments. Lentil seedling harvested on 16 February 2016. Total duration was required 95 days from sowing to harvesting time of lentil crops. During harvesting, three samples of lentil pods were randomly collected separately from each plot and tagged with proper marking of each sample. Then samples were dried at room temperature. Next, all samples were dried in an oven at 55°C for 72 hours towards the digestion of samples for the determination of As uptake in lentil’s grains from As contaminated field soils.

### Experiment 3: Reduction of arsenic uptake through AMF

#### Lentil genotypes

Based on the pervious field experiments, BARI Mashur 1 and BARI Mashur 5 were selected for the reduction of As uptake through AMF inoculation. These pot experiments were conducted in a green house with a controlled environment at BSMRAU.

#### Arbuscular Mycorrhizal Fungus (AMF)

AMF species were collected from International Culture Collection of (Vesicular) Arbuscular Mycorrhizal Fungi (INVAM), West Virginia University (WV), USA. AMF samples were mixed with soils and roots of the host plant of Sorghum that was housed in the Department of Environmental Science at BSMRAU. Mixture of soil and roots were collected from this cultured area as a source of AMF. Based on the total biomass (soil) in the pots, 4% AMF content soils were used for the reduction of As uptake in lentil crops. Mycorrhizas spores in the soil and root samples were observed by following the Wet Sieving and Decanting Method [28,29]. About 70–100 spore was found in each kg soils. The colonization of AMF species was found 40–60% with the host plant of sorghum root.

#### Growing of lentil genotypes in pot soils

The 8mgkg^-1^ As concentrated BJRI soils was taken for growing of lentil plants. This As concentration in background soils (8 mgkg^-1^) was increased to 45 mgkg^-1^ through the addition of sodium arsenate (Na_2_HAsO_4_.7H_2_O) as a source of arsenic. A 0.154 g sodium arsenate was used for each kg of soil in the pots to get the required concentration of arsenic (45 mgkg^-1^). 1200g ground soils kept in each pot for growing lentil. Recommended doses of fertilizers such as, Urea 225kgha^-1^, TSP 450kgha^-1^ and MOP 175 kgha^-1^ were applied to each pot. BARI Mashur 1 and BARI Mashur 5 was sown on 13^th^ April 2016 in a controlled temperature greenhouse at BSMRAU. Temperatures ranged from 18°C to 20°C in the greenhouse for growing of lentil genotypes in this pot experiment.

#### Treatments and replications

Two lentil genotypes- BARI Mashur 1 and BARI Mashur 5 were selected and treatments were T_1_ = 8 mgkg^-1^ arsenic concentration in soils, and T_2_ = 45 mgkg^-1^ arsenic concentrated soils. A 150 g of soil with root mixture as Arbuscular Mycorrhizal Fungi (AMF) used for the reduction of As uptake in root and shoot of lentil crops. Five replications with two lentil varieties were used in both AMF and non-AMF soils and the total number of pot was 40.

#### Shoot length, root and shoot mass in AMF and non- AMF soils

Randomly, average shoot length measured through measuring tape (cm) at week 4 in each treated pots. During this week, five lentil plants harvested from As treated each pot. Average fresh weight of root and shoot taken separately through an electrical balance (g) in AMF and non-AMF treated experiment. Similarly, average dry weight of root and shoot of lentil plants measured independently during this week. All samples were dried in an oven at 55°C for 72 hours towards the digestion for the analysis of As uptake reduction in root and shoot of lentil genotypes.

#### Digestion of samples

Soils, lentil roots, shoots and grains were digested separately following heating block digestion procedure [30]. Of the soil/compost samples, 0.2 g taken into clean, dry digestion tubes and 5 ml of concentrate HNO3 and 3 ml concentrate HCLO4 added to it. The mixture was allowed to stand overnight under fume hood. In the following day, this vessel put into digestion block for 4 hours at 120^0^ C temperature. Similarly, 0.2 g ground root, shoot and grains samples put into clean digestion vessel and 5 ml concentrate HNO3 added to it. The mixture was allowed to stand overnight under fume hood. In the following day, this vessel put into digestion block for 1 hours at 120^0^ C temperature. This content cooled and 3 ml HCLO4 added to it. Again, samples put into the heating block for 3–4 hours at 140°C. Generally heating stopped whenever a white dense fume of HCLO4 emitted into air. Then samples cooled, diluted to 25ml with de-ionized water and filtered through Whatman No 42 filter paper for soil and plant samples. Finally, samples were stored with polyethylene bottles. Prior to samples digestion, all glassware was washed with 2% HNO3 followed by rinsing with de-ionized water and drying.

#### Analysis of total arsenic

Digested samples were brought into the laboratory of Bangladesh Council of Scientific and Industrial Research (BCSIR) for the analysis of As concentration in lentil root, shoot, -grains, soil, and vermi-compost. The concentration of As in root, shoot, grains of lentil plants, soil, vermi-compost and water samples were analyzed by flow injection hydride generation atomic absorption spectrophotometry (FI-HG-AAS, Perkin Elmer A Analyst 400, USA) using external calibration [31]. The optimum HCl concentration was 10% v/v and 0.4% NaBH_4_ produced the maximum sensitivity. Three replicates taken from each digested samples and the mean values obtained based on the calculation of those three replicates. Standard Reference Materials (SRM) from National Institute of Standards and Technology (NIST), USA analyzed in the same procedure at the start, during and at the end of the measurements to ensure continued accuracy.

#### Statistical analysis

The design of the experiment was followed Completely Randomized Block (CRD). Analysis of Variance (ANOVA), interaction between treatments and varieties, means comparison of treatments and varieties, treatment- varieties and soils interaction on arsenic uptake with reduction using AMF in lentil roots, shoots and grains were analyzed using software R.

## Results

### Chemical properties of lentil seed, soil and water samples

The ranges of dry weight of lentil seeds were 9.43 to 9.53 g of 10g BARI released lentil genotypes. Among all lentil cultivars, BARI Mashur 1, BARI Mashur 5, BARI Mashur 6, and BARI Mashur 7 seeds were found As free. The highest As concentration (0.05mgkg^-1^) was found in the seeds of BARI Mashur 4. The distilled water was As free as well as 0.02 mgl^-1^ concentrated As were present in irrigation water. The ranges of pH found 6.75 to 7.93 in vermi-compost, BSMRAU, BJRI and Mathchar soils. The percentages of total nitrogen, available phosphorus, exchangeable potassium, and available sulfur were detected 1.23%, 57.71, 150 and 698.04 mgkg^-1^ in vermi-compost samples, accordingly. As well, the total nitrogen, available phosphorus, exchangeable potassium, and available sulfur were detected 0.057%, 14.41, 120 and 9.615 mgkg^-1^ in BJRI soil samples, separately. Similarly, in BSMRAU soil samples, the total nitrogen, available phosphorus, exchangeable potassium, and available sulfur were found 0.11%, 20.68, 124 and 23.07 mgkg^-1^, respectively. On the other hand, Mathchar soil samples content 0.086% of total nitrogen, 9.177 mgkg^-1^ available phosphorus, 128 mgkg^-1^ exchangeable potassium, and 2.884 mgkg^-1^ available sulfur. Total As concentration found 2.688, 8.299, 5.223, and 14.633 mgkg^-1^ in vermi-compost, BJRI, BSMRAU, and Mathchar soil samples, respectively (Table 1).

### Arsenic reduce shoot length, root and shoot mass of lentil genotypes

**Fig 1:**
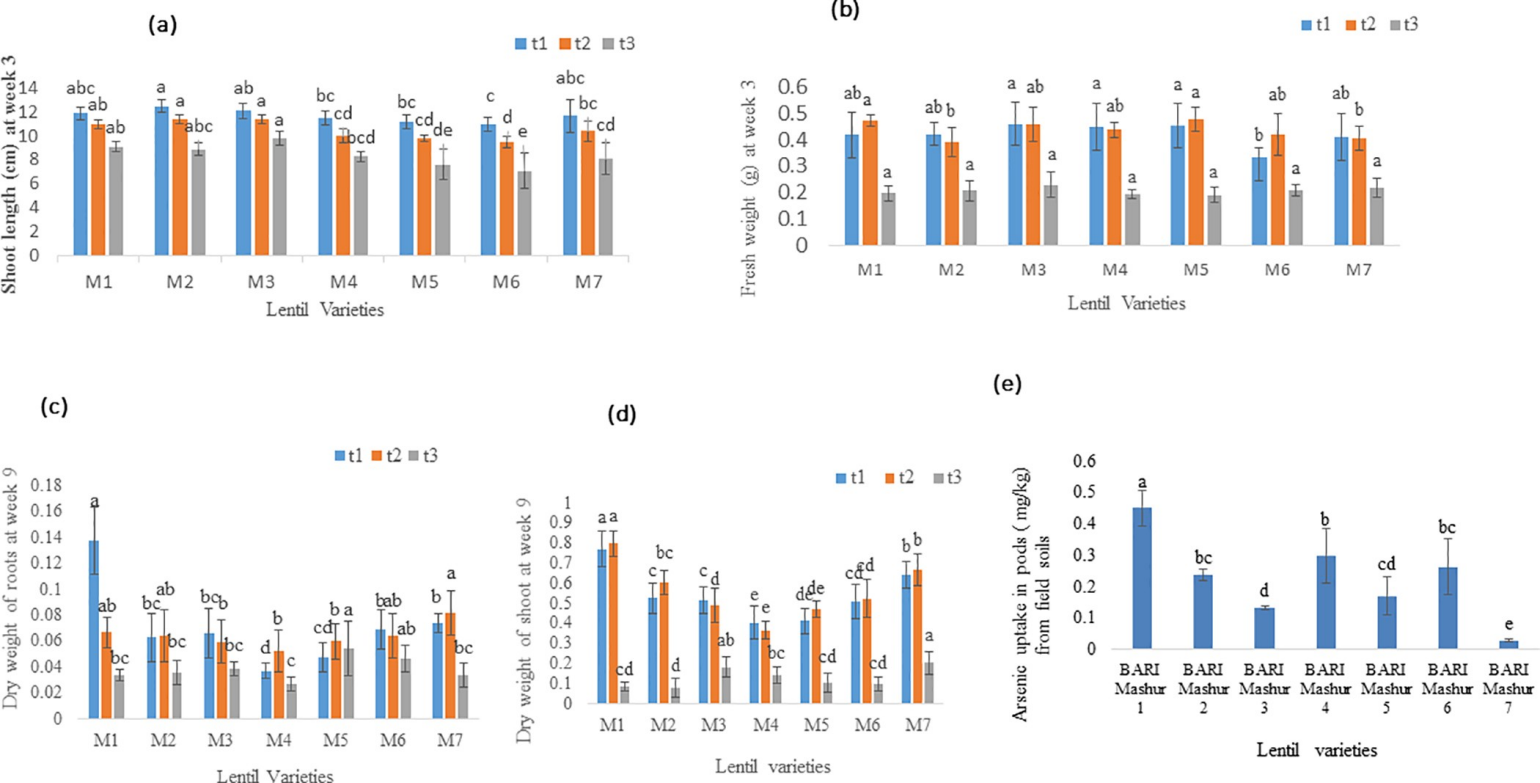
**Effect of arsenic uptake on a) shoot length (Mean ± SEM), b) fresh weight (Mean± SEM) of lentil varieties at week 3; c) dry weight of roots (Mean ± SEM), d) dry weight of shoots (Mean ± SEM) of lentil varieties at week 9; e) Average arsenic accumulation in lentil grains (Mean ± SEM) from arsenic contaminated field soils**. Means denoted by different letters under the same As level indicate significant difference at 0.1% level of significance. M_1_, M_2_, M_3_, M_4_, M_5_, M_6_ and M_7_ indicate BARI Mashur 1, BARI Mashur 2, BARI Mashur 3, BARI Mashur 4, BARI Mashur 5, BARI Mashur 6, & BARI Mashur 7, lentil genotypes, respectively. Arsenic treatments indicate concentration of arsenic in soil; t1, control, 5mg arsenic kg^-1^ soil; t2, 15 mg arsenic kg^-1^ soil; t3, 100 mg arsenic kg^-1^ soil.

The highest average shoot length of BARI Mashur 2, BARI Mashur 2 &3, and BARI Mashur 3 was 12.5, 11.4, and 9.8 (cm) in t_1_ (arsenic concentration 5 mg in each kg soils), t_2_ (arsenic concentration 15 mg in each kg soils) and t_3_ (arsenic concentration 100 mg in each kg soils) treated lentil seedlings at week 3, respectively. Shoot length of t_3_ treated in BARI Mashur 6 lentil were found significantly lower (*p< 0*.*001)* than other lentil seedlings (Fig 1A). The fresh weight (0.182–0.20 g) was not significantly increased (*p< 0*.*001)* in t_3_ treated Lentil seedlings. The lowest fresh weight 0.189g was found in t_3_ treated BARI Mashur 5 lentil seedlings at week 3 (Fig 1B). Dry weight of root and shoot were recorded comparatively lower in t_3_ treated lentil seedling than t_1_ and t_2_. Dry weight of roots in t_3_ treated BARI Mashur 5 lentil genotype was found significantly different from other lentil genotypes. Meanwhile, dry weight of shoot in t_3_ treated BARI Mashur 7 was found significantly higher than BARI Mashur 1, 2, 4, 5 and 6 lentil genotypes at week 9 (Fig 1C and 1D).

### Arsenic uptake in root and shoot of lentil genotypes

Treatments effect on the arsenic uptake in root and shoot of lentil genotypes were found statistically significant (*p<0*.*001)* different. Interaction effect of varieties and treatments on As uptake in lentil roots (*p< 0*.*001)* were found significantly different (Table 2). Mean comparison between treatment 1 & 2 (*0*.*001≤p<0*.*01*), 1 & 3(*p< 0*.*001)*, and 2 &3 (*p< 0*.*001)* for As uptake in roots were found significantly different. As well, the mean comparison of treatment 1 & 3 and treatment 2 & 3 both were found statistically identical (*p< 0*.*001*) on As uptake in lentil shoot (Table 3). Interaction of BARI Mashur 1&3 (*0*.*01≤p<0*.*0*.*05*), BARI Mashur1 & 4 (0.05≤p<0.0.1), BARI Mashur1 & 5 (*0*.*001≤p<0*.*01*), BARI Mashur1 & 6 (0.01≤p<0.05), BARI Mashur 2 & 3 (*p<0*.*001*), BARI Mashur 2 & 4 (*0*.*001≤p<0*.*01*), BARI Mashur 2 & 5 (*p< 0*.*001*), BARI Mashur 2 & 6 (*0*.*001≤p<0*.*01*), and BARI Mashur 2 & 7 (*0*.*01≤p<0*.*05*) were found statistically significant on As uptake in their roots (Table 3). The mean comparison of the interaction between treatments and lentil varieties on As uptake in their roots were found significantly (*p< 0*.*001*, *0*.*001≤p<0*.*01*, *0*.*01≤p< 0*.*05*) different (Table 3).

**Table 2:** ANOVA on Arsenic uptake in root and shoot of lentil grown in As contaminated soils.

Source of variations (SV)	Degrees of freedom (DF)	Arsenic uptake in root			Arsenic uptake in shoot		
		Sum of Squares (SS)	Mean Sum of Squares (MSS)	F value	Sum of Squares (SS)	Mean Sum of Squares (MSS)	F value
Variety	6	472	78.67	4.225***	151	25.17	1.607
Treatment	2	18870	9435	507.001***	12647	6323.5	404.976***
Variety : Treatment	12	756	63	3.387***	303	25.25	1.617 ^**NS**^
Residuals	84	1563	18.607		1312	15.619	

*** indicates significant difference at *p<0*.*001* level of significance

**Table 3:** Arsenic uptake in root and shoot of lentil genotypes.

Treatment interaction	Arsenic in root	Arsenic in shoot	Interaction of varieties	Arsenic in root
-	-	-	BARI Mashur 1 & BARI Mashur 3	-3.73317*
-	-	-	BARI Mashur 1 & BARI Mashur 4	-2.81283 ^**(.)**^
-	-	-	BARI Mashur 1 & BARI Mashur 5	-4.86325**
-	-	-	BARI Mashur 1 & BARI Mashur 6	-3.39101*
-	-	-	BARI Mashur 1 & BARI Mashur 7	-2.2915
-	-	-	BARI Mashur 2 & BARI Mashur 3	-5.45291***
Treatment 1 & 2	-3.31224**	-0.9072943	BARI Mashur 2 & BARI Mashur 4	-4.53258**
Treatment 1 & 3	-29.949***	-23.7215229***	BARI Mashur 2 & BARI Mashur 5	-6.58299***
Treatment 2 & 3	-26.6368***	-22.8142286***	BARI Mashur 2 & BARI Mashur 6	-5.11076**
Treatment: Variety -Treatment: Variety			Arsenic in root	
t3:BARI Mashur1-t1: BARI Mashur1			24.7691***	
t3: BARI Mashur 2-t1:BARI Mashur1			18.8576***	
t3:BARI Mashur 3-t1:BARI Mashur 1			35.60782***	
t3:BARI Mashur 4-t1:BARI Mashur1			30.74864***	
t3:BARI Mashur 5-t1:BARI Mashur 1			36.92746***	
t3:BARI Mashur 6-t1: BARI Mashur1			32.77742***	
t3:BARI Mashur 7-t1:BARI Mashur1			29.89708***	
t3:BARI Mashur1-t2: BARI Mashur1			22.88976***	
t3:BARI Mashur 2-t2: BARI Mashur 1			16.97826***	
t3:BARI Mashur3-t2: BARI Mashur1			33.72848***	
t3: BARI Mashur 4-t2: BARI Mashur 1			28.8693***	
t3: BARI Mashur 5-t2: BARI Mashur 1			35.04812***	
t3: BARI Mashur 6-t2: BARI Mashur 1			30.89808***	
t3: BARI Mashur 7-t2: BARI Mashur 1			28.01774***	
t2: BARI Mashur 2-t3: BARI Mashur 1			-22.1289***	
t2: BARI Mashur 3-t3: BARI Mashur 1			-22.3117***	
t2: BARI Mashur 4-t3: BARI Mashur 1			-20.376***	
t2: BARI Mashur 5-t3: BARI Mashur 1			-20.3782***	
t2: BARI Mashur 6-t3: BARI Mashur 1			-20.6582***	
t2: BARI Mashur 7-t3: BARI Mashur 1			-21.513***	

*** indicates significant difference at *p<0*.*001* level of significance

** indicates significant difference at *0*.*001≤p<0*.*01* level of significance

* indicates significant difference at *0*.*01≤p<0*.*05* level of significance

^**(.)**^ Indicates significant difference at *0*.*05≤p< 0*.*1* level of significance

### Arsenic uptake in pod of lentil varieties from As contaminated field soils

The collected of BARI released seven lentil varieties were cultivated in 5 mgkg^-1^ As contaminated field soils. Among these varieties, BARI Mashur 1 was the highest As (0.45 mgkg^-1^) accumulator and the lowest As (0.029 mgkg^-1^) accumulator was BARI Mashur 7 in its grain. On the other hand, an average As concentration found 0.237, 0.133, 0.298, 0.17, and 0.262 mgkg^-1^ in pods of BARI Mashur 2, BARI Mashur 3, BARI Mashur 4, BARI Mashur 5, and BARI Mashur 6, respectively. Arsenic was significantly increased in grains of BARI Mashur 1 lentil in compare to other genotypes (Fig 1E).

### Effect of AMF on biomass production of lentil genotypes in As contaminated soils

**Fig 2:**
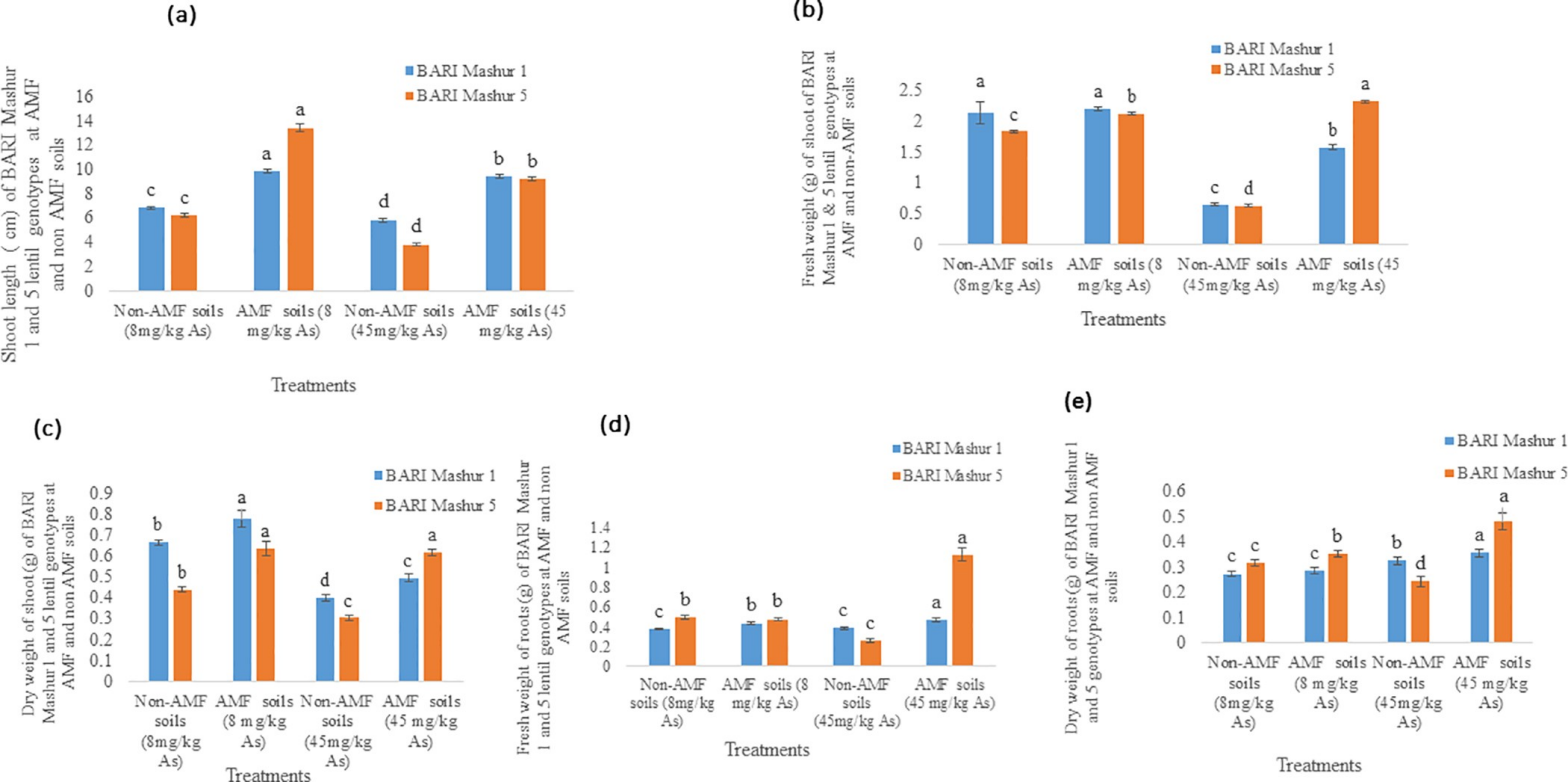
**Effect of Arbuscular Mycorrhizal Fungus (AMF) on a) shoot length (Mean± SEM), b) fresh weight of shoot (Mean ± SEM), c) dry weight of shoot (Mean± SEM), d) fresh weight of roots (Mean ± SEM), e) dry weight of roots (Mean± SEM) at arsenic treated BARI Mashur 1 & 5 lentil genotypes.** Means denoted by different letters under the same As level indicate significant difference at 0.1% level of significance.

In Non- AMF soils, shoot length of BARI Mashur 1 and BARI Mashur 5 were 6.8 and 6.2 cm in T_1_ (arsenic concentration 8 mg in each kg soils) treated lentil seedlings. Shoot, length of lentil was 5.8, and 3.8 cm were in BARI Mashur 1, and BARI Mashur 5 at T_2_ (arsenic concentration 45 mg in each kg soils) treated seedlings. AMF treated shoot length in BARI Mashur 1 and 5 at 8 mgkg^-1^ and 45 mgkg^-1^ arsenic concentrated both soils were found significantly higher than non AMF soils during week 4 (Fig 2A). Fresh and dry weight of shoot both were found significantly lower in non-AMF treated 45 mgkg^-1^ As concentrated soils at week 5 (Fig 2B and 2C). As well as, AMF has significant effect for the increasing of dry and fresh weight of root in lentil genotypes (Fig 2D and 2E).

### AMF reduce As uptake in root and shoot of lentil genotypes

Treatment & varietal interaction effect on the reduction of As uptake in root and shoot of lentil genotypes at AMF soil were found statistically significant (*p<0*.*001)*. Likewise, AMF had significant effect on the reduction of As uptake in both treated lentil genotypes (Table 4A). Mean comparison effects of the interaction between treatments and varieties *(p<0*.*001)* on the reduction of As uptake in the root and shoot mass of lentil genotypes at AMF applied soils were found significantly different (Table 4B). According to ANOVA, interaction effect between treatment, varieties & soils on the reduction of As uptake in shoot mass was found significantly different (*p<0*.*001)* (Table 5A). Similarly, As uptake significantly reduce in shoot of lentil genotypes (*p<0*.*001; 0*.*001≤p<0*.*01)* according to the interaction between treatments, varieties and AMF soils (Table 5B).

**Table 4:** AMF reduce As uptake in root and shoot of lentil genotypes.

A							
Source of variations (SV)	Degrees of freedom (DF)	As in root at non-AMF soil			As in shoot at non-AMF soil		
		Sum of Squares (SS)	Mean Sum of Squares (MSS)	F value	Sum of Squares (SS)	Mean Sum of Squares (MSS)	F value
Treatment	1	2422.8	2422.8	1909.596***	398.9	398.9	1772.89***
Variety	1	120.2	120.2	94.739***	53.2	53.2	236.44***
Treat: Variety	1	112.7	112.7	88.828***	27.8	27.8	123.56***
Residuals	16	20.3	1.26875		3.6	0.225	
		AMF reduce As uptake in root			AMF reduce As uptake in shoot		
Treatment	1	1380.0	1380.0	869.29***	153.00	153.00	807.92***
Variety	1	92.4	92.4	58.21***	13.25	13.25	69.97***
Treat: Variety	1	83.3	83.3	52.47***	5.50	5.50	29.04***
Residuals	16	25.4	1.5875		3.03	0.189375	
B							
Treatment: Variety-Treatment : Variety				As in root at non-AMF soil	As in shoot at non- AMF soil	AMF reduce As uptake in root	AMF reduce As uptake in shoot
T_2_ : BARI Mashur 1- T_1_ : BARI Mashur 1				26.7598***	6.5734***	20.6952***	4.4832***
T_1_ : BARI Mashur 5- T_1_ : BARI Mashur 1				-0.1566	0.9032^.^	-0.2166	0.5796
T_2_ : BARI Mashur 5- T_1_ : BARI Mashur 1				17.1094***	12.1932***	12.3146***	7.1598***
T1 : BARI Mashur 5- T2 : BARI Mashur 1				-26.9164***	-5.6702***	-20.9118***	3.9036***
T_2_ : BARI Mashur 5- T_2_ : BARI Mashur 1				-9.6504***	5.6198***	-8.3806***	2.6766***
T_2_ : BARI Mashur 5- T_1_ : BARI Mashur 5				17.266***	11.29***	12.5312***	6.5802***

*** indicates significant difference at *p<0*.*001* level of significance

^**(.)**^ Indicates significant difference at *0*.*05≤p< 0*.*1* level of significance

**Table 5:** AMF reduce As uptake in root and shoot of lentil genotypes according to the interaction between treatments, varieties and soils.

A							
Source of variations (SV)	Degrees of freedom (DF)	As in root			As in shoot		
		Sum of Squares (SS)	Mean Sum of Squares (MSS)	F value	Sum of Squares (SS)	Mean Sum of Squares (MSS)	F value
Treatment	1	3730	3730	2594.78***	523.0	523.0	2497.91***
Variety	1	212	212	147.48***	59.8	59.8	285.61***
Soil	1	129	129	89.74***	46.5	46.5	222.09***
Treat: Variety	1	195	195	135.65***	29.0	29.0	138.51***
Treat: Soil	1	73	73	50.78***	28.9	28.9	138.03***
Variety : Soil	1	1	1	0.696	6.7	6.7	32.02***
Treatment: Variety: Soil	1	1	1	0.696	4.3	4.3	20.54***
Residuals	32	46	1.4375		6.7	0.209375	
B							
Treatment: Variety: Soil- Treatment: Variety: Soil							AMF reduces As uptake in shoot
T_2_: BARI Mashur 1: non AMF—T_1_: BARI Mashur 1: non AMF soil							6.5734***
T_1_: BARI Mashur 5: non AMF—T_1_: BARI Mashur 1: non AMF soil							0.9032
T_2_: BARI Mashur 5: non AMF—T_1_: BARI Mashur 1: non AMF soil							12.1932***
T_2_: BARI Mashur 1: AMF—T_1_: BARI Mashur 1: non AMF soil							4.1892***
T_1_: BARI Mashur 5: AMF—T_1_: BARI Mashur 1: non AMF soil							0.2856
T_2_: BARI Mashur 5: AMF—T_1_: BARI Mashur 1: non AMF soil							6.8658***
T_1_: BARI Mashur 5: non AMF–T_2_: BARI Mashur 1: non AMF soil							-5.6702***
T_2_: BARI Mashur 5: non AMF–T_2_: BARI Mashur 1: non AMF soil							5.6198***
T_1_: BARI Mashur 1: AMF—T_1_: BARI Mashur 1: non AMF soil							-6.8674***
T_2_: BARI Mashur 1: AMF–T_2_: BARI Mashur 1: non AMF soil							-2.3842***
T_1_: BARI Mashur 5: AMF–T_2_: BARI Mashur 1: non AMF soil							-6.2878***
T_2_: BARI Mashur 5: non AMF—T_1_: BARI Mashur 5: non AMF soil							11.29***
T_1_: BARI Mashur 1: AMF—T_1_: BARI Mashur 5: non AMF soil							-1.1972***
T_2_: BARI Mashur 1: AMF—T_1_: BARI Mashur 5: non AMF soil							3.286***
T_2_: BARI Mashur 5: AMF—T_1_: BARI Mashur 5: non AMF soil							5.9626***
T_1_: BARI Mashur 1: AMF–T_2_: BARI Mashur 5: non AMF soil							-12.4872***
T_2_: BARI Mashur 1: AMF–T_2_: BARI Mashur 5: non AMF soil							-8.004***
T_1_: BARI Mashur 5: AMF–T_2_: BARI Mashur 5: non AMF soil							-11.9076***
T_2_: BARI Mashur 5: AMF–T_2_: BARI Mashur 5: non AMF soil							-5.3274***
T_2_: BARI Mashur 1: AMF—T_1_: BARI Mashur 1: AMF soil							4.4832***
T_2_: BARI Mashur 5: AMF—T_1_: BARI Mashur 1: AMF soil							7.1598***
T_1_: BARI Mashur 5: AMF–T_2_: BARI Mashur 1: AMF soil							-3.9036***
T_2_: BARI Mashur 5: AMF–T_2_: BARI Mashur 1: AMF soil							2.6766***
T_2_: BARI Mashur 5: AMF—T_1_: BARI Mashur 5: AMF soil							6.5802***

*** indicates significant difference at *p<0*.*001* level of significance.

## Discussion

Arsenic (As) contamination in food chains has been reported in many countries throughout the world, with the most severe problems found in Asia, particularly in Bangladesh [32,33]. In Bangladesh, the contamination of As in groundwater was confirmed in 1993 [34]. Since then, this contamination has been extended to crop fields due to the irrigation of ground water [34,35]. Among several contaminated areas, Faridpur region is one of the severely As-contaminated sites in Bangladesh. Most of these areas became As polluted due to extensive uses of ground water for irrigation in the crop fields. We found about 15 mgkg^-1^ As in background soils of these regions, this concentration is definitely toxic for the development of root, shoot and grains for many cereal crops as well as lentil plants (Table 1). Similarly, As contamination in food crops is also highly visible in other region of Bangladesh including West Bengal, India [36,37].

Lentil is an important edible leguminous crop in Bangladesh and a rich source of plant-based proteins, essential for physiological growth of human beings. Nevertheless, food crops including rice and lentil are being contaminated due to presence of high concentrations of As in soils. Generally, lentil is grown in dry season, and thus irrigation is needed for successful cultivation of this crop. When lentil is grown in As-contaminated fields, As present in background soils and irrigation water accumulates in lentil roots, shoots and grains [4]. The uptake of As in root, shoot and grains of lentil crops is connected with the changes in several nutrient in soils specially phosphate content in soils [4,38]. We found that phosphorus concentration (9–57 mgkg^-1^) in soil samples for pot experiment could increase the As accumulation in lentil roots, shoots and grains (Table 1).

Arsenic accumulation significantly affected plant biomass accumulation in lentil genotypes (Fig 1A–1D). In addition, different vegetative indexes such as root length, shoot height, root and shoot mass of lentil plants were studied in the present experiment (Fig 1A–1C). [39] reported the sensitivity of vegetative response in the following order: root length>root mass>shoot length>total mass (root + shoot)>shoot mass>germination. Accordingly, we also found that As sensitivity was higher in lentil roots, followed by shoots, and pods. Shoot height, plant biomass (root + shoot + pod) and root length were significantly affected with increasing As concentrations in soils. For instance, total biomass of lentil crops was found to be in more jeopardy in 100 mgkg^-1^ As concentrated soils than other treated pots (5 mgkg^-1^ As; 15 mg kg^-1^ As) of lentil seedlings (Fig 1B–1D).

Lentil is the number one pulse crop and a rich source of protein. It is also one of the cheapest sources of plant proteins. Lentil pods, roots and shoots are used as food for humans, and animals throughout the world. Arsenic has been identified as a non-threshold human carcinogen [40]. Arsenic transportation to edible plant parts as well as food chain contamination is conditional on the availability of As in soils from its source. The Bengal Delta Plain is considered to be the largest mass poisoning in the history of humanity as millions of people are exposed and suffered from the effects of chronic As intoxication [41]. The concentrations of As in the groundwater in Bangladesh and West Bengal (India) exceed by several times the permissible levels set internationally and nationally [42,43]. As Bangladesh is the second largest As-contaminated region in the world, ensuring As free food crops for human consumption remains a key challenge in this region. All lentil varieties released by BARI are promising varieties in Bangladesh and the world as well. However, before this study, no systematic experiment was conducted to explore As uptake from soil to root, shoot and grain in these lentil genotypes of Bangladesh. Despite less genotypic variations, all lentil varieties showed significant differences in As accumulation in their roots in 5, 15 and 100 mgkg^-1^ concentrated soils. We found that all lentil varieties were in good conditions during seedling stage in 5 and 15 mgkg^-1^ As concentrated soils compared to the 100mgkg^-1^ concentrated soils (Fig 1B–1D). A significant concentration of As was transported from soils to lentil pods in the current study. Notably, BARI Mashur 1 genotype accumulated a higher concentration of As (0.45 mgkg^-1^) in grains than that in other genotypes (Fig 1E). Similarly, irrespective of As dose, roots accumulated higher concentrations of As than shoots and grains. In agreement with our results, higher As concentrations in roots were reported by [44,45,46] and [47] in food crops. There are, however, no previous reports of elevated As concentrations in lentil grains. Thus, this research has significant importance in terms of human food chain contamination through consumption of As-contaminated lentil.

The universal colonization with arbuscular mycorrhizal fungus (AMF) can alleviate multiple abiotic stresses in a range of plant species [16,19, 48]. While low As accumulator lentil genotypes are important for human consumption, AMF could reduce the As uptake in roots, shoots and grains of lentil crops [18]. AMF colonized with lentil roots deter As uptake and reduce As toxicity through the symbiotic relationship between each other [23]. Due to the reduction of As toxicity, plants generally show increases in growth compared with Non-AMF controls grown at the same As and P supplies in soil [44, 49,50,51,52]. We found that BARI Mashur 1 and BARI Mashur 5 both lentil genotypes performed better for their growth of roots and shoots in 8 mgkg^-1^ and 45 mgkg^-1^ As concentrated AMF applied soils than non-AMF. Shoot length, root and shoot mass of lentil were found higher in AMF-treated As-contaminated soils. In other words, growth of roots and shoots was satisfactory in both varieties of lentil when mutually treated with AMF in As-contaminated soils (Fig 2A–2E).

In the current study, As concentration increased significantly in roots and shoots of BARI Mashur lentil genotype (Table 3). We also provide convincing evidence that AMF can reduce As uptake in root and shoot in lentil genotypes (Tables 4B and 5B). Research also showed that AMF have their substantial effect on plant growth [16,19, 45]. The growth parameters of BARI Mashur 1 and 5 lentil genotypes increased significantly with the application of AMF in As concentrated soils (Fig 2A–2E). It emphasized that AMF inoculation reduced As translocation from soil to plant and increased growth and nutrient uptake and chlorophyll content of food crops [53]. Similarly, there is growing evidence that mycorrhizal fungi might alleviate As toxicity in the host plant by acting as a barrier in soils [54]. It has been widely reported that mycorrhizal fungi can increase the tolerance of their host plants to heavy metals when present at toxic levels [55,56]. Consistently, [23,57,58] demonstrated that, at high levels of As concentrations in soils, AMF infection reduced the concentration of As in plant biomass.

Plant growth changes due to the presence of toxic substances and availability of nutrient in soils [59]. Arsenic toxicity is one of the important limiting factors for the nutrient availability in soils, which directly deterred to stunt of plant growth. For this, it is important to improve soil health conditions through the mitigation of As toxicity in soils. Here, we used AMF for the improvement of soil condition particularly for the mitigation of As toxicity. There is also evidence that AMF can be effective in 8 and 45 mgkg^-1^ As concentrated soils for the reduction of As uptake in root and shoot from soils (Tables 4B and 5B). In tomato, AMF colonization increases biomass as well as reduces As uptake in roots and shoots in 100 to 500 mgkg^-1^ As concentrated soils [53]. AMF not only colonize the root cortex but also extend the network of hyphae into the surrounding environment. These external hyphae can contribute to improving plant nutrients for increasing the biomass growth as well as can alleviate heavy metal toxicity by modulating the metal acquisition in plants from contaminated soils [60]. Since most of the cations are essential, complete exclusion is not possible and selective efflux would be more likely. That’s why, arsenate uptake by the hyphae of AMF (*R*. *irregularis)* occurs via the high-affinity phosphate transporter GiPT [61,62]. However, the direct involvement of arbuscular mycorrhizal fungi (AMF) in detoxification mechanisms still remain largely unclear. Soils treated with AMF show that fungal colonization could dramatically increase plant biomass accumulation [63]. Moreover, a positive effect of mycorrhizal inoculation on growth of lentil (*L*. *culinaris*), P nutrition, and attenuation of As toxicity has also been reported in plant soil interaction [63]. Reduced uptake of As by lentil roots and subsequently, attenuated translocation to shoots and grains will reduce risk associated with the consumption of As-contaminated food. Thus, lentil crops grown in As contaminated soils with AMF colonization can potentially reduce As entry into human body through food chains.

## Conclusion

Arsenic is the number one carcinogenic substance. Among 37 countries affected by As, Bangladesh is the second largest As-contaminated country in the world. Not only Bangladesh, many other countries have also been identified hugely contaminated with this toxic and hazardous substance. Lentil is one of the most important legume crops in Bangladesh as well as throughout the world due to its high protein content. Therefore, this protein source should be free from any potential toxin for human beings. Reduction of As in food as well as mitigation of As phyto-toxicity in lentil genotypes is significantly important to meet the future demand of safe food. Here, we found that BARI Mashur 1 lentil genotype is a high As accumulator genotype compared to the other released lentil varieties in Bangladesh. AMF application decreased As concentrations in roots and shoots of lentil genotypes. We also found that AMF could effectively reduce As transport from soils to roots, shoots and grains of lentil plants. These results clarified the important role of AMF in mitigation of As uptake in root shoot and reallocation to grains of lentil genotypes, which might have important implications in supplying toxin free lentil grains in As-affected areas of the world.

## Supporting information

S1 Dataset Effect of As on biomass growth of lentil genotypes. (XLSX)

S2 Dataset Arsenic accumulation in lentil grains from field soils. (XLS)

S3 Dataset AMF improve biomass growth in lentil genotypes grown in As soil. (XLSX)
